# Reliability agreement in foul and penalty judgements between officials in the Swedish hockey league

**DOI:** 10.3389/fspor.2024.1425040

**Published:** 2024-12-16

**Authors:** Glenn Björklund, Olivia Procter, Mikael Swarén

**Affiliations:** ^1^ Department of Health Sciences, Swedish Winter Sports Research Centre, Mid Sweden University, Östersund, Sweden; ^2^ Swedish Unit for Metrology in Sports, School of Health and Welfare, Dalarna University, Falun, Sweden

**Keywords:** ice hockey, sports, SHL, Fleiss' kappa, inter-rater agreement

## Abstract

**Introduction:**

Officials are essential in terms of player safety and injury prevention, especially in contact team sports such as ice hockey, where numerous fast pace and high force contacts occur. If against the rules, these collisions can result in penalties. However, there is limited literature on the inter-rater reliability of the officials’ decisions. Hence, the purpose was to investigate the theoretical reliability agreement between professional ice hockey officials in the Swedish Hockey League (SHL).

**Method:**

Fifty video clips with different match situations were shown to 33 professional ice hockey officials in the SHL. Each situation was shown three times and the officials had 20 s between each video clip to answer which offence and penalty they would judge. The answers were anonymously collected using an online questionnaire. Fleiss’ kappa was used to assess the reliability agreement between the referees, for each situation.

**Results:**

The Fleiss’ kappa values for all officials were 0.63 and 0.35 for offences and penalties, respectively. Referees and linesmen had similar kappa values for offences (0.64 vs. 0.64), as well as for penalties (0.38 vs. 0.35).

**Conclusion:**

The results show that the suggested methodology can be used to identify situations where officials agree and disagree. In ice hockey, poor agreement regarding penalties can depend on the chosen offence as the rulebook limits the availability of penalties, based on the chosen offence. This can create issues, as there are situations where different offences are equally correct but will result in different penalties.

## 1 Introduction

Officials and referees are a critical part of the success of sport. They need to deliver correct decisions in a time pressured, dynamic environment that directly impacts the current play, and ultimately the competition outcome (1). With a steep increase of economic interest into sport, alongside increased media and commercialization, incorrect decisions can be detrimental to not only the match outcome but to wider prospects such as careers and financial repercussions (2, 3). Therefore, referee behavior must be assessed.

Through the years, many governing bodies have faced scrutiny from the media about officiating quality and therefore a growth of literature has occurred across a range of sports; football, rugby, basketball, gymnastics, figure skating and ice hockey, to name a few (4). Primarily, research revolves around influences of external factors and biases that might cause incorrect officiating decisions (5). Situational aspects such as home advantage, crowd noise, and international/national bias are the main focus (6–11). Psychological explanations such as gaze behavior and attentional bias (12–14), level of competition (15), and individual/team characteristics (16, 17) are also well researched. A top level soccer referee makes about 137 observable decision per match, and it can be speculated that the total number of decisions to be around 200 per match (18). However, in team sports specifically, referees appear to “balance penalties” and often change their behavior dependent on the context and timing of the game, and that the “foul standard” can be unique to each game (5, 19, 20). For example, Anderson & Pierce (5) found that NCAA basketball referees were more likely to call a foul on the team with the fewest fouls, keeping it even throughout the game. This coincides with a study by Burnett et al. (21) where umpires in English netball super-league were found to give fewer decisions as the match progressed with an average of 33 in quarter 1 and 27 in quarter 4. Similarly, in rugby league the occurrence of penalty judgements has been found to drop within the last 10 min of the match to not disrupt play (19).

For instance, Mascarenhas et al. (22) highlighted how shared mental models improve the coherence of decisions, particularly in dynamic environments like rugby. Similarly, Bruno et al. (23) demonstrated that discrepancies in decision thresholds among handball referees, where individual interpretations of fouls vary, can lead to inconsistencies, reducing the fairness of officiating. Fuller et al.'s (24) analysis of football referees showed that inconsistencies in assessing fouls, especially those leading to injuries, can jeopardize player safety. Werger et al. (25) also highlight the imperative role of officials in ensuring player safety and preventing injuries. This is especially critical in contact team sports such as ice hockey where numerous fast pace, high force player and equipment contacts occur (26). Icing and off sides have been reported as “easy to call”, however decisions become more complex and variable when deciding and distinguishing penalties in response to undesirable behavior (27). Physical tactics such as body checking are frequently used to slow/stop opponents. These are difficult to distinguish, and consequently are a high cause of injury (28).

In a study by Ackery et al. (29), 40% of 632 Canadian referees said that injuries in ice hockey were due to the game and players becoming too aggressive. However, in a prior study by Tegner and Lorentzon (30), only 8% of 285 injuries across two seasons were backed up by a penalty call, therefore suggesting referees definitely have a crucial role to play in reducing aggressive play and increasing the safety of the game. If limited penalties are given in response to a foul, a team's ability to push the rules to the limit increases (31, 32). Furthermore, with ambiguous rules regarding fouls, contextual factors can grow influentially, creating a lack of cohesion between referee decisions (33). Alternative practices can develop between groups and therefore potentially cause misjudgments. If officials cannot be unanimous in theory, then it is unlikely they will come to cohesive decisions in a live situation with the additional pressures and external biases.

Previous literature within ice hockey has focused on situational aspects facing officials’ decisions (8, 34, 35). These include monitoring match play i.e., the timing of foul calls (27, 36), and recognizing that foul calls are influenced by team tactics and strategy dependent on the score, i.e., more “conservative play” when they have already incurred multiple penalties (37). To improve foul calls, studies have also researched the utilization of practice videos to enhance decision making of novice officials as well as investigating the reliability agreement for head contact situations, among officials in youth leagues (38, 39). However, as mentioned by Russel et al. (20), it is important that officials practice making decisions based on context and that positively influence each game's trajectory. But there is still important for a league, such as the Swedish Hockey League (SHL) with an average of six penalties per game during the 2021–2022 season, to know the inter-official agreement and the individual decision thresholds in order to ensure consistency, fairness, and accuracy in officiating (23, 40). Hence, with limited literature on the inter-rater reliability of the officials’ decisions (41), this study aims to investigate how theoretically unanimous officials’ decisions are within the Swedish Hockey League.

## 2 Method

### 2.1 Participants

In total, 33 professional officials (14 referees and 19 linesmen) from the Swedish Hockey League (SHL) participated in the study. The study was approved by the institutional review board at Mid Sweden University, Östersund, Sweden. All data were anonymously collected in a questionnaire, without any personal information and as part of the participating officials’ ordinary work description. All participants had been given oral and written information about the study prior to the data collection and gave their written consent to participate. The study was conducted in accordance with the Declaration of Helsinki.

### 2.2 Protocol

The officials were presented with 50 video clips depicting various scenarios from professional ice hockey games. These clips were carefully selected by a panel comprising three staff members from the SHL situation room, one representative from the International Ice Hockey Federation (IIHF) officiating group, and three officiating directors from the SHL and the Swedish Ice Hockey Federation. The selection process began with the pool of over 1,500 situations analysed each season by the SHL situation room. However, not all these scenarios involve offences, the database includes all scored goals, potential goals, verbal offences, and other situations requiring objective analysis. From this extensive pool, the panel selected situations that included both offences and non-offences. The selection criteria prioritised representation of offences and penalties outlined in the rulebook, video clarity, and the independence of the action (ensuring the situation was standalone rather than influenced by or part of a preceding event). The result was a curated set of 50 clips designed to test officiating consistency and decision-making.

Each situation was shown three times and the officials had 20 s between each video clip to answer which offence and penalty they would judge, Tables 1, 2. The average length of each clip was 9.6 ± 2.7 s. Officials were seated apart from each other, and all communication was prohibited during the session. The answers were anonymously collected using an online questionnaire (surveymonkey.com) and the data exported for further statistical analyses.

**Table 1:** List of offences.

Abuse of officials	Physical abuse of officials	Boarding	Charging
Elbowing	Fighting	Head-butting	Illegal check to the head
Kicking	Kneeing	Roughing	Slew-footing
Checking from behind	Holding	Hooking	Interference
Tripping	Butt-ending	Cross-checking	High-sticking
Slashing	Spearing	Delaying the game	Diving/embellishment
Equipment	Handling puck	Illegal substitution	Interference of the goalkeeper
Leaving the bench	Refusing to start play	Too many players on the ice	Unsportsmanlike conduct
Face-offs	Goals	High-sticking the puck	Line change
Off-side	Puck out of bounds	Start of game and periods	Illegal hit
Specific equipment rules	Throwing equipment	No penalty	

**Table 2:** List of penalties.

Minor	Double minor
Bench minor	Major—without automatic game misconduct
Major with automatic game misconduct	Misconduct penalty
Game misconduct	Match penalty
Penalty shoot	No penalty

### 2.3 Statistical analysis

The inter-rater reliability (agreement between the referees) for each situation was measured with Fleiss’ kappa (42). The kappa value provides practical information regarding the agreement among multiple raters, which simplifies the practical interpretation and implementation of the results, as suggested by Johnson et al. (43). The interpretation of the Fleiss’ kappa value are based on Landis and Koch (44), and presented in Table 3. All statistical analyses were performed using jamovi (45).

**Table 3:** Interpretation of fleiss’ kappa.

< 0.00	Poor agreement
0.0–0.20	Slight agreement
0.21–0.40	Fair agreement
0.41–0.60	Moderate agreement
0.61–0.80	Substantial agreement
0.81–1.0	Almost perfect agreement

## 3 Results

The Fleiss’ kappa values for all officials were 0.63 and 0.35 for offences and penalties, respectively. Referees and linesmen had similar kappa values for offences (0.64 vs. 0.64), as well as for penalties (0.38 vs. 0.35).

**Figure 1:**
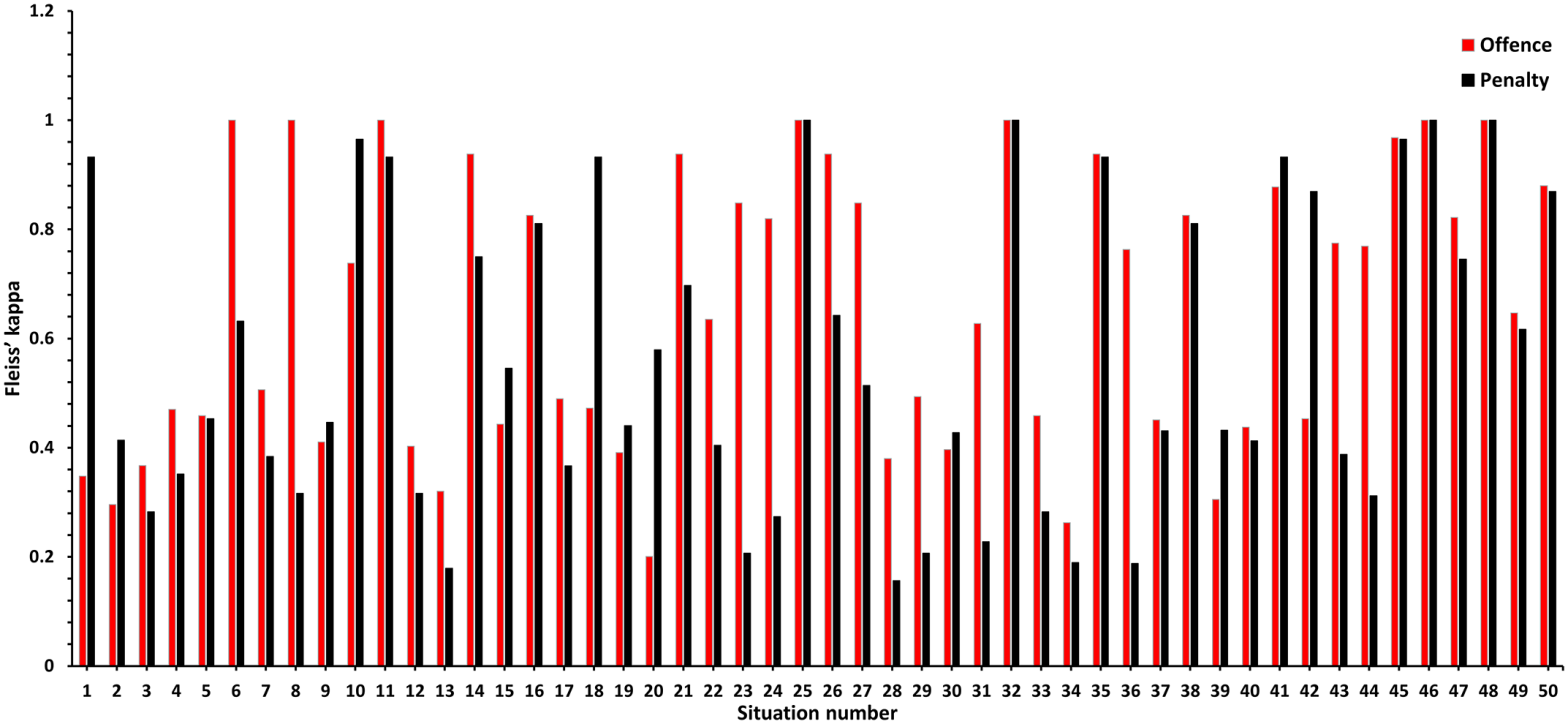
Calculated kappa values for offence and penalty for each video situation.

The specific kappa value for each video situation is presented in Figure 1.

Frequency analyses of the offences and penalties for the situations with a kappa value <0.4 are presented in Tables 4, 5.

**Table 4:** Frequency analysis of the offence situations with fair agreement or less (kappa value <0.40).

	Situation number							
	1	2	3	13	20	28	34	39
Boarding				14				
Charging				3		19		
Head-butting				1				
Holding	16	10			1			
Hooking	12	1			8			
Kneeing								11
Interference	4	6		1	6		2	1
Illegal check to the head			18	13		5		
Cross-checking			10					
Illegal hit						1		
Slashing		1					1	
Tripping					10		3	5
Slew-footing							15	
High-sticking			2				9	
Diving								1
No penalty	1	15	3		8	7	3	15

**Table 5:** Frequency analysis of the penalty situations with fair agreement or less (kappa value <0.40).

	Situation number												
	3	8	12	13	23	24	28	29	31	33	34	36	44
Minor	19	3	19	3	9	2	14	8	15	17	10	12	13
Double minor			3			2					2		
Bench minor	1		1	9		0	1			1	1		
Major without automatic game misconduct	1	8	2	13	7	1	4	3	2	6		1	3
Major with automatic game misconduct	5	19	8		14	10	4	6	10	9	3	7	16
Misconduct penalty				1									
Game misconduct	1	1		7		1		1	1			1	
Match penalty	3	2			3	17	3	15			14	11	1
Penalty shot													
No penalty	3						7		5		3	1	

The analysis shows that several of the situations with low level of agreement for the offence and/or penalty, often can be clustered together in groups that have similar attributes, e.g., situation 1 with either holding or hooking, situation 3 with either cross-checking or illegal check to the head or situation 13 with either boarding, charging or illegal check to the head. The situations with low agreement are mainly for situations with offences, resulting in either major with or without game misconduct and match penalty.

## 4 Discussion

The present results show a higher level of agreement between the officials regarding offences compared to penalties (kappa value 0.63 vs. 0.35). Even though linesmen are not responsible for deciding offences or penalties on the ice, they still had similar kappa values as the referees for both offences and penalties. The agreement among officials is an important factor in ice hockey as well as in several other sports, e.g., gymnastics, ski jumping, figure skating, slope style and mogul skiing, as athletes, coaches and spectators all want fair competitions as well as there are strong economic incentives to have fair and accurate officials (9). However, to best of the authors’ knowledge, this is the first paper that assesses the reliability agreement between professional sports officials. The participating officials were all professional officials, working full time in the Swedish Ice Hockey League (SHL), which is considered as one of the best ice hockey leagues in the world. The present study investigated the agreement between professional ice hockey officials, but the presented methodology could be used and implemented in several other sports as well.

**Figure 2:**
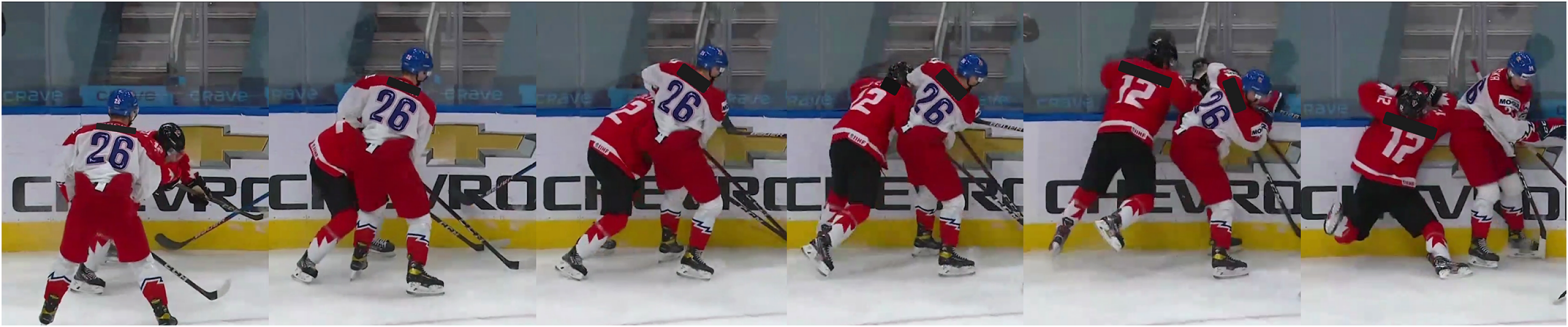
Key images from situation 13 where either checking from behind, boarding, charging or illegal check to the head can be considered correct.

As seen if Figure 1, situations 1, 18 and 20 have much lower kappa values for offences compared to the kappa values for penalties. Based on the type of offence and situation, some offences can be clustered together into the same group. For example, the different offences roughing, cross-checking, and checking from behind can all meet the criteria to be handed out in the same situation. It is up to the referee to decide which offence and the justified penalty for it, even though it can be argued that all the three offences are correct. This is shown in Figure 2, showing key images from situation 13 where the agreement between the officials was low. Here, the officials could correctly rule either checking from behind, boarding, charging or illegal check to the head, which most officials also did (Table 4). Hence, the kappa value for a specific situation can be negatively affected when several different types of offences can be correct. Therefore, care must be taken when interpreting the reliability agreement between referees for these kinds of situations.

It can be argued that the outcome of the game is not majorly affected by which offence is handed out, as long as the ruled penalty is consistent and correct. However, according to the ice hockey rule book (46), different offences result in different penalties. For example, “illegal check to the head” is either a minor or a major with game misconduct, whereas “charging” can result in any of the available penalties except a double minor penalty. Another example is “checking from behind,” which does not include the “major without game misconduct” penalty, whereas the “boarding” offence includes it. As a result, officials can be forced to choose an offence that aligns with the penalty they deem appropriate, rather than selecting the offence most representative of the situation. This practice introduces a layer of subjectivity to penalty calls, as officials are constrained by the penalty-offence link outlined in the rules. Moreover, the limitation of video review to major offences further restricts officials’ ability to deliberate and deliver the most appropriate penalty for a situation. Officials must decide in real-time whether to escalate a penalty to access video review, potentially leading to calls that prioritise procedural requirements over factual accuracy. During a game, this system will contribute to lower levels of agreement on penalties, as noted in the results.

Our findings, as illustrated in Figure 1, demonstrate instances where the agreement on offences is near-perfect, but the penalties show only fair or even slight agreement (e.g., situations 6, 8, 23, and 24). This disparity suggests that while officials may agree on the categorisation of offences, the subsequent penalties reveal variability in the interpretation of severity or appropriateness. In these cases, the span of penalties, from no penalty to a match penalty, reflects a lack of consensus that can significantly impact the game's dynamics. Such large discrepancies introduce unpredictability, potentially eroding the trust of players and coaches in the officiating process. The implications of these disagreements extend beyond the immediate game outcomes. For players, inconsistent penalty decisions can create confusion about what is permissible on the ice, potentially increasing the risk of injury due to uncertain expectations about enforcement. This ambiguity can also undermine the players’ ability to adapt their actions to avoid offences and their associated consequences, heightening the physical risk inherent in a high-speed and contact-intensive sport like ice hockey.

From the perspective of spectators, visible disagreements among officials can detract from the viewing experience and amplify frustration, potentially leading to increased aggression toward both officials and players. This can create an aggressive environment that negatively impacts the overall atmosphere of the game, as well as the mental well-being of officials, increasing the officials’ intentions to quit (47, 48). Moreover, a heightened perception of inconsistency in officiating may diminish the perceived fairness of the sport, which is crucial not only for maintaining fan engagement and the sport's reputation but also for meeting the expectations of athletes and coaches who seek fair competition. Additionally, as Heiniger and Mercier (9) point out, there are strong economic incentives to ensure fair and accurate officiating, as the credibility of the sport and its ability to attract spectators and sponsors heavily depend on the trustworthiness of its officials.

It must be noted that the methodology used in the present study only accounts for the officials’ theoretical judgment of each shown situation. In a real game, the officials consider numerous other aspects of each situation, e.g., previous actions, the nature and intensity of the game as well as trying to achieve perceptions of balance and fairness while also being affected by the crowd (8, 27). The current study does not take this into consideration and should hence be considered as an assessment of the unanimousness of the referees’ theoretical interpretation of the IIHF rule book. Still, this is important as it is fair to assume that if the officials do not agree about the theoretical interpretation of the rule book, it is unlikely that they have a better practical agreement on the ice during games. A further limitation of the present study is the selection of situations. The SHL situation room views thousands of situations each season which makes it difficult to objectively select situations that represent all aspects of the game. However, the 50 situations in the study were selected by professional and experienced staff, who considered the situations to be representative of difficult situations in the SHL. Williams et al. (39) used the same methodology, as the current study, to analyse the reliability to detect head impact among Level II-III referees in Canada. Their findings (39) are in line with our results with Fleiss’ kappa revealing fair to moderate agreement between raters, and that the lowest agreement was for penalty intensity. Willamson et al. (39) also compared the referees’ rulings to a gold standard, consisting of two high performance referees. Hence future studies with professional officials should also investigate the reliability agreement between officials on the ice and the officials in the situations room and from the IIHF. In addition, it can be hypothesised that future projects might benefit focusing on specific types of offences and/or penalties to get more robust statistics and applicable results.

## 5 Conclusions

The results from the current study show that the suggested methodology of using Fleiss’ kappa to assess the reliability agreement between ice hockey officials provides valuable and useful information about the unanimousness of the referees’ theoretical interpretation of the IIHF rule book. However, future assessments of the unanimousness among sports referees could benefit from using e.g., “type of offence” instead of naming the exact offence. This might reduce the risk of low kappa values due to open situations where different offences could be equally correct. Still, one could argue that the IIHF rule book has too many different offences, forcing the referees to be too detailed in their judgments on the ice which can result in unfair time penalties for the players. This is enhanced by the fact that all offences cannot be combined with all penalties, which makes the choosing of the specific offence unnecessarily important.

## Data Availability

The datasets presented in the present article are not readily available as the participants did not consent to share the data outside of the research team. Requests to access the data should be directed to miv@du.se.
